# Residual current detection method based on improved VMD-BPNN

**DOI:** 10.1371/journal.pone.0289129

**Published:** 2024-02-08

**Authors:** Yunpeng Bai, Xiangke Zhang, Yajing Wang, Lei Wang, Qinqin Wei, Wenlei Zhao

**Affiliations:** 1 School of Electrical and Electronic Engineering, Shandong University of Technology, Zibo, Shandong, China; 2 Institute of Electrical Engineering, Chinese Academy of Sciences, Haidian District, Beijing, China; 3 Shandong Institutes of Industrial Technology, Jinan, Shandong, China; 4 Shandong Linglong Tyre Limited Company, Zhaoyuan, Shandong, China; Univerzitet Singidunum, SERBIA

## Abstract

To further enhance the residual current detection capability of low-voltage distribution networks, an improved adaptive residual current detection method that combines variational modal decomposition (VMD) and BP neural network (BPNN) is proposed. Firstly, the method employs the envelope entropy as the adaptability function, optimizes the [k, ɑ] combination value of the VMD decomposition using the bacterial foraging-particle swarm algorithm (BFO-PSO), and utilizes the interrelation number R as the classification index with the Least Mean Square Algorithm (LMS) to classify, filter, and extract the effective signal from the decomposed signal. Then, the extracted signals are detected by BPNN, and the training data are utilized to predict the residual current signals. Simulation and experimental data demonstrate that the proposed algorithm exhibits strong robustness and high detection accuracy. With an ambient noise of 10dB, the signal-to-noise ratio is 16.3108dB, the RMSE is 0.4359, and the goodness-of-fit is 0.9627 after processing by the algorithm presented in this paper, which are superior to the Variational Modal Decomposition-Long Short-Term Memory (VMD-LSTM) and Normalized-Least Mean Square (N-LMS) detection methods. The results were also statistically analyzed in conjunction with the Kolmogorov-Smirnov test, which demonstrated significance at the experimental data level, indicating the high accuracy of the algorithms presented in this paper and providing a certain reference for new residual current protection devices for biological body electrocution.

## Introduction

Residual current operated protective devices (RCD) are important safety devices widely used in rural low-voltage grids. However, the presence of numerous interfering signals mixed with the residual current during grid faults can significantly reduce the detection accuracy of RCD, leading to frequent instances of false tripping and non-tripping. [1]. In recent years, with the rapid development of modern signal processing technology, many domestic and foreign scholars have proposed a variety of methods to improve the accuracy and speed of residual current detection, such as wavelet analysis, Empirical Mode Decomposition (EMD), neural networks [2,3] and other algorithms have been applied to residual current detection, combining these emerging technologies with RCD to further improve the reliability of RCD. For example, X. Xiao et al. proposed the adaptive filtering detection method [4], which achieves adaptive current detection by noise reduction and residual current separation of the measured current, but the N-LMS model has the disadvantages of too slow convergence and low detection accuracy; H. Yan et al. proposed the EMD detection method [5], which uses the characteristics of the time-frequency domain change of residual current as the basis for determining the occurrence of electrocution faults, which is better improved accuracy, but the detection is prone to problems such as modal mixing and endpoint effects; C. Li et al. performed the detection of residual current by combining VMD with LSTM [6], which has certain advantages due to LSTM in sequence modelling problems with long-time memory. Therefore, it can accurately handle large sample data and improve detection accuracy. However, LSTM increases the time span and increases the computational effort when dealing with larger magnitude sequences; S. Wu proposed a residual current detection model based on wavelet entropy, using Auto Encoder to extract feature information and BPNN (WE-AE-BP) for classification to achieve the detection and classification process of residual currents [7].WE needs to set up the fundamental wave, decomposition layer in advance, threshold and threshold function, which reduces the adaptability of the algorithm. X. Xiong proposed a residual current detection model based on sample entropy [8], which extracts the BESC (Bioelectric shock characteristic) to determine whether the electrocution is applied, but strong noise and high frequency harmonics can greatly interfere with the experimental results.

Previous studies have shown that modern signal processing techniques can be used for residual current detection and have achieved good results. However, there are still some drawbacks, such as the need for manual parameter settings in methods like WE, EMD and VMD, which greatly affect the accuracy of the data. Additionally, the BESC and LMS methods perform poorly in high-noise environments and have lower accuracy. In this paper, VMD is combined with BFO-PSO to adaptively select parameters, reducing the randomness of manual parameter settings and improving the accuracy of detection. This provides a data foundation for future research directions, such as combining machine learning and big data analysis, for residual current prediction and classification [9].

BP neural networks have the advantages of having fewer weight parameters and stable parallel processing. By back-propagating the errors, the weights and biases of the neurons in each layer are iteratively updated, which greatly improves the accuracy of residual current detection. Therefore, this paper uses BPNN for residual current detection. In practical applications, the collected residual current signal is usually mixed with a large amount of noise, which affects the accuracy of detection. Considering that VMD is sensitive to noise and can effectively handle non-linear and non-stationary signals, VMD is chosen to pre-process the residual current signal. Unlike previous works, the number of decomposition layers and penalty factor of VMD need to be set in advance. In this paper, a combination of the Bacterial Foraging-Particle Swarm Algorithm is proposed to adaptively select the optimal value of VMD decomposition layers and penalty factor. To solve the problem of inaccurate information extraction caused by the aliasing effect that may exist in VMD, the IMF is divided into mixed and noisy components by introducing the mutual relation number R [10]. Due to its simplicity, efficiency, and ease of implementation, LMS has been chosen to further extract information from the mixed components in order to enhance the accuracy of the information. Building upon this, a residual current detection model was constructed by combining the BP neural network, which was used to predict the residual current signal. This paper provides a statistical analysis of the obtained data, demonstrating the relevance and accuracy of the data. It serves as a reference for the study of a novel residual current protection device based on biological body electrocution.

In the second chapter of this paper, the structural model of the BP neural network is introduced, while the third chapter describes the VMD method for noise reduction of residual current signals. By combining BFO-PSO to optimize the number of decomposition layers and penalty factor, an adaptive VMD model is constructed. In the fourth section, further information extraction of the noise-reduced residual current signal is performed using R-LMS. The fifth chapter presents the improved VMD-BPNN model and algorithm flow. The sixth and seventh chapters respectively verify the effectiveness of the algorithm through human simulation experiments and actual data collection and analysis.

## Detection principle of the BPNN

**Fig 1:**
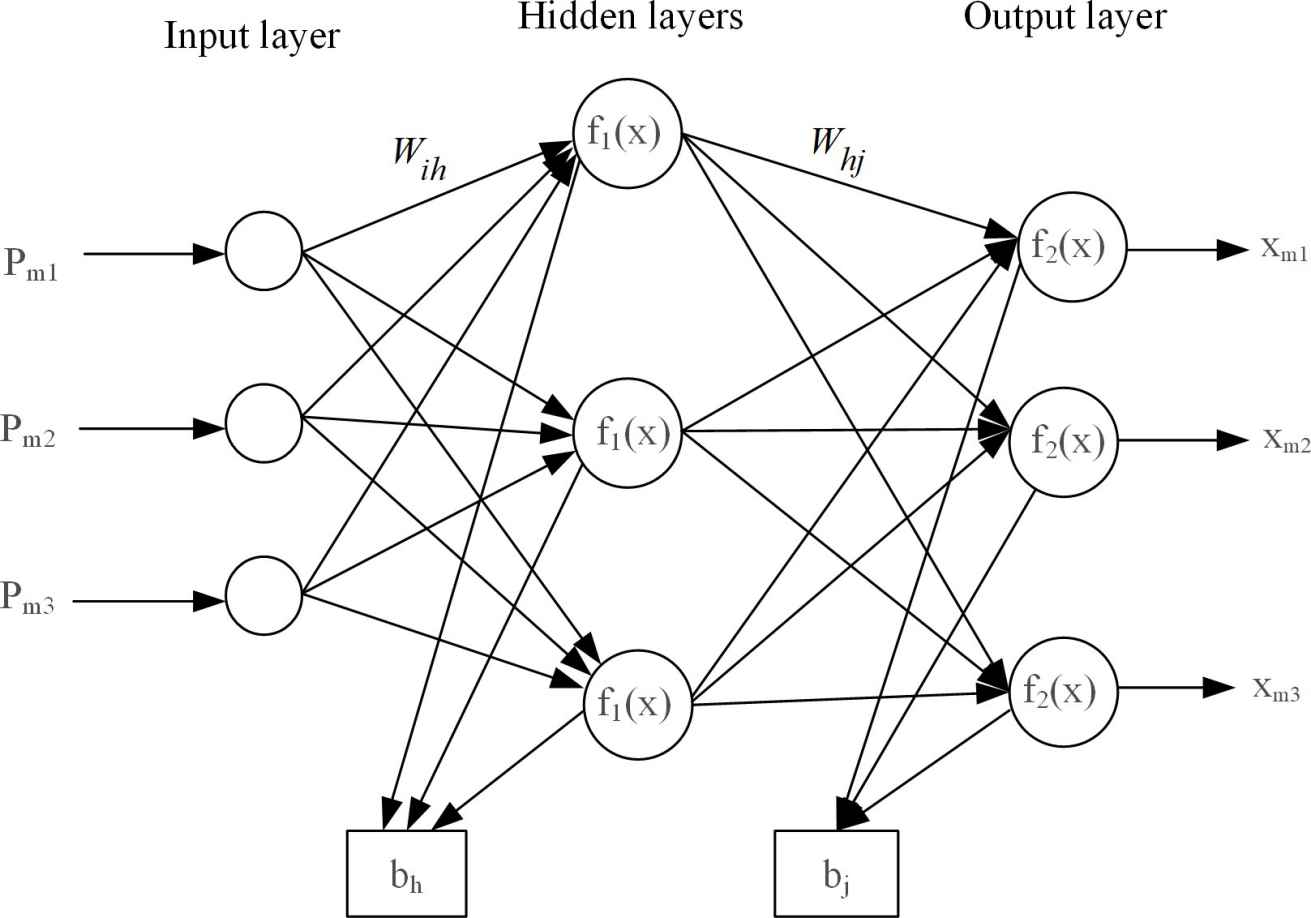
BPNN topology diagram.

The BP neural network is a multi-layer feedforward neural network that combines forward signal transmission and backward error propagation for residual current detection. This paper uses a 3-layer neural network as an example to explain the detection principle [11]. The detection structure of BPNN is shown in Fig 1. The structure of BPNN consists of three parts: the input layer, the hidden layer, and the output layer. The training process consists of two stages: forward propagation and backward propagation. Firstly, the input signal is denoted as pm, and the output detection value is denoted as *x*_*m*_. The input layer receives the residual current signal *P*_*m*_, and the residual current signal is propagated through each layer. The output of the *h*_*th*_neuron in the hidden layer is shown in Eq (1), and the activation functions of the hidden layer and the output layer are denoted as*f*_1_(*x*) and *f*_2_(*x*), respectively, as shown in Eq (2).


$$\alpha_{h}=f_{1}(\sum_{i=1}^{3}W_{ih}P_{m}+b_{h})$$ (1)

$$f_{1}(x)=f_{2}(x)=\frac{e^{P_{m}}-e^{P_{m}}}{e^{P_{m}}+e^{P_{m}}}$$ (2)

Where *W*_*ih*_ and *b*_*h*_ are the connection weights and thresholds from the input layer to the hidden layer, respectively. *α*_*h*_ is the output value of the *h*_*th*_ neuron in the hidden layer. *P*_*m*_ is the input residual current signal,

After the calculation in the hidden layer, the forward flow continues to the output layer, resulting in the computed output *x*_*m*_. At this point, the forward propagation stage is completed. Assuming that the number of neurons in the hidden layer is q, the output of the *j*_*th*_ neuron in the output layer is shown in Eq (3).


$$x_{m}=f_{2}(\sum_{h=1}^{q}W_{hj}\alpha_{h}+b_{j})$$ (3)

Where *W*_*hj*_ and *b*_*j*_ are the connection weights and thresholds from the hidden layer to the output layer, respectively.

During the process of backward propagation, if the computed output *x*_*m*_ from the output layer has a significant error compared to the desired output, the error signal between the target output and the computed output needs to be propagated backward and the interlayer weights continuously adjusted during the iteration process. The error function and weight update formula are shown in Eqs (4) and (5), respectively. This process continues until the training error or the number of training iterations reaches the preset limit. After adjustment through the backward propagation process, the computed *x*_*m*_ from the output layer becomes the final output detection value.


$$E=\frac{1}{2}{\left\|x_{m}-P_{m}\right\|}^{2}$$ (4)

$$\Delta W=\eta \frac{\partial E}{\partial W}$$ (5)

Where E represents the error function, *x*_*m*_ represents the detection output value, *P*_*m*_ represents the residual current input value, *η* represents the learning rate, and *W* represents the interlayer weights.

## Improved VMD noise reduction

In practice, the residual current signal collected usually contains a large amount of noise, which affects the detection accuracy of BPNN, so the actual measured residual current signal should be pre-processed. Variational Mode Decomposition (VMD) can effectively avoid the problems of mode mixing and boundary effects, and has the advantage of good noise immunity [12,13]. Therefore, this paper uses VMD to pre-process the residual current signal.

### The principle of VMD

In the VMD algorithm, the intrinsic mode function (IMF) is defined as a bandwidth -constrained amplitude-modulation function, and the original signal is decomposed into a specified number of IMF components by constructing and solving a constrained variational problem. The constraint for constructing the variational problem is given in (6).


$$\{\begin{array}{l} \underset{\left\{u_{k}(t),\omega_{k}(t)\right\}}{\min}\{\sum_{k}{\left\|\partial_{t}[\left(\delta (t)+\frac{j}{\pi t})*u_{k}(t\right)]e^{-j\omega_{k}t}\right\|}_{2}^{2}\}\\ s.t.\sum_{k=1}^{k}u_{k}(t)=x(t) \end{array}$$ (6)

Where *x*(*t*) is the original noisy signal, *u*_*k*_(*t*) is the kth modal component of *x*(*t*),and k is the number of decompositions of IMFs, k = 1,2,…,k. *δ*(*t*) is the impulse function, *j* is the imaginary unit, * denotes the convolution, *ω*_*t*_ is the central frequency of the IMF, and the constraint is that the modal sum is equal to the input signal. In order to solve (6), a quadratic penalty factor *α* and a Lagrange multiplier operator *λ* can be introduced to convert (6) into an unconstrained variational problem, and the extended Lagrange function is expressed as (7).


$$L\left(\{u_{k}\},\{\omega_{k}\},\lambda \right)=\alpha {\sum_{k}\left\|\partial_{t}[\left(\delta (t)+\frac{j}{\pi t})*u_{k}(t\right)]e^{-j\omega_{k}t}\right\|}_{2}^{2}+{\left\|f(t)-\sum_{k}u_{k}(t)\right\|}_{2}^{2}+\langle \lambda (t),f(t)-\sum_{k}u_{k}(t)\rangle$$ (7)

Using the alternating multiplier algorithm to alternately update $u_{n+1}^{k}$、 $\omega_{n+1}^{k}$ and $\lambda_{n+1}$, and solve for the optimal solution of Eq (7), the solution iteration process is described in the literature [14], which leads to expressions (8) 、(9)and(10).

$${\hat{u}}_{k}^{n+1}(\omega )=\frac{\hat{f}(\omega )-\sum_{l<k}{\hat{u}}_{l}^{n+1}(\omega )-\sum_{l>k}{\hat{u}}_{l}^{n}(\omega )+\frac{{\hat{\lambda }}^{n}(\omega )}{2}}{1+2\alpha {\left(\omega -\omega_{k}^{n}\right)}^{2}}$$ (8)

$$\omega_{k}^{n+1}=\frac{\int_{0}^{\infty }\omega {\left|{\hat{u}}_{k}^{n+1}(\omega )\right|}^{2}d\omega }{\int_{0}^{\infty }{\left|{\hat{u}}_{k}^{n+1}(\omega )\right|}^{2}d\omega }$$ (9)

$${\hat{\lambda }}^{n+1}(\omega )={\hat{\lambda }}^{n}(\omega )+\tau (\hat{f}(\omega )-\sum_{k=1}^{k}{\hat{u}}_{k}^{n+1}(\omega ))$$ (10)

where ${\hat{u}}_{l}^{n}(\omega )$、 $\hat{f}(\omega )$、 ${\hat{\lambda }}^{n}(\omega )$ and ${\hat{u}}_{k}^{n+1}(\omega )$ represent the Fourier transforms of $u_{l}^{n}(\omega )$、 *f*(*ω*)、 *λ*^*n*^(*w*) and $u_{k}^{n+1}(\omega )$ respectively; *τ* is update parameters, and n is the number of iterations. The convergence condition of the iterations is shown in Eq (11).

$$\frac{\sum_{k+1}^{k}{\left\|{\hat{u}}_{k}^{n+1}-{\hat{u}}_{k}^{n}\right\|}_{2}^{2}}{{\left\|{\hat{u}}_{k}^{n}\right\|}_{2}^{2}}<\varepsilon_{1}$$ (11)

where *ε*_1_ is the predetermined convergence error.

### Optimisation of VMD parameters based on BFO-PSO

The number of decomposition layers K and the penalty factor α in the VMD algorithm need to be chosen artificially [15,16]. Too large a choice of K values tends to result in over-decomposition, while too small a choice results in modal conflation and loss of information. while the size of α will affect the bandwidth size of the components and affect the balance of the VMD process. Literature [1,10] proposed the instantaneous power averaging method and the interrelationship number judgment method to determine the k-value, respectively, However, all of the above methods have the disadvantage of being computationally intensive and are not suitable for time-sensitive signal processing processes. In recent years, metaheuristic optimisation algorithms have attracted much attention by virtue of their ability to quickly and accurately solve optimisation problems with multivariate functions [17,18], such as the beetle antennae search approach [19], Hybrid Moth Flame Optimization [20], etc. They can be combined with different requirements and targeted to provide specific solutions to different problems, and in this paper, the bacterial foraging particle swarm algorithm is chosen to solve the problem of adaptive parameter selection for VMD. In this paper, the basic idea of particle swarm algorithm is introduced into the bacterial foraging algorithm to construct a hybrid bacterial foraging optimization algorithm, which has good search speed and accuracy, can effectively make up for the defects of slow BFO operation and low accuracy of PSO operation, avoid the problem of local convergence, and is suitable for solving the optimization of complex functions [21]. Bacterial Foraging Optimization-Particle Swarm Optimization (BFO-PSO) has the advantages of fast global search and high operational accuracy compared to similar population intelligence algorithms such as Fruit Fly Optimization Algorithm (FOA) [22] and Ant Colony Optimization (ACO) [23], so this paper proposes the bacterial particle swarm optimization algorithm for the combination of parameters of VMD [*k*, ɑ] for simultaneous optimization search. The Particle Swarm finds the individual optimal position *p*_*best*_ and the population optimal position *g*_*best*_ by updating its velocity and position, and the function iteration terminates when both are in the same position. During the iteration, the Particle Swarm updates its own velocity and position as follows:

$$V_{i}^{k+1}=\omega V_{i}^{k}+c_{1}r_{1}(P_{i}^{k}-x_{i}^{k})+c_{2}r_{2}(P_{g}^{k}-X_{i}^{k})$$ (12)

$$X_{i}^{k+1}=X_{i}^{k}+V_{i}^{k+1}$$ (13)

Where *ω* is the inertia weight, *c*_1_,*c*_2_ is the acceleration factor, *r*_1_,*r*_2_ is a random number between [0,1]. *k* is the number of iterations, $P_{i}^{k}$ is the historical optimal solution searched by the *i*_*th*_ particle up to the *k*_*th*_ generation, $P_{g}^{k}$ is the optimal solution searched by the whole particle population up to the kth generation, $X_{i}^{k}$, $V_{i}^{k}$ are the current position and flight speed of the particle respectively.

BFO is optimized primarily through three behaviors: convergence, replication and dispersal, where bacteria are driven together by the release of repulsive and gravitational signals between them, where the fitness function and position update equations are shown in Eqs (14) and (15).

$$J(i,j,k,l)=J(i,j,k,l)+J_{cc}[\theta^{i}(j,k,l)+P(j,k,l)]$$ (14)

$$\theta^{i}(j+1,k,l)=\theta^{i}(j,k,l)+C(i)\varphi (j)$$ (15)

where *θ*^*i*^(*j*,*k*,*l*) and *P*(*j*,*k*,*l*) are the position of the bacteria in the *j*_*th*_ convergence in the *k*_*th*_ replication of the *l*_*th*_ migration process and the position of each bacteria in the population, respectively, *C*(*i*) is the randomly chosen step size in [–1,1] that determines the position at the (*j*+1)_*th*_ convergence after one step in the direction, *j*_*cc*_ denotes the interbacterial attraction of the *i*_*th*_ bacteria, and *φ*(*j*) represents the randomly chosen unit direction vector after rotation。

By using Formulas (12) and (13) of PSO algorithm to simplify position update in BFO, BFO algorithm can accelerate to find the optimal solution in the replication process, increase the global search ability in the dispersal process, and speed up the search for the optimal objective function. The improved formula is as follows:

$$\theta^{i}(j+1,k,l)=X_{i}^{k}+[\omega V_{i}^{k}+c_{1}r_{1}(P_{i}^{k}-x_{i}^{k})+c_{2}r_{2}(P_{g}^{k}-X_{i}^{k})]\varphi (j)$$ (16)

Considering that entropy not only responds to the uniform characteristics of the probability distribution, but also evaluates the sparse characteristics of the signal, the minimum value of the envelope entropy is chosen as the fitness function [13], where the adaptability function is as in (17)

$$\left\{ \begin{array}{l} E_{e}=-\sum_{i=1}^{N}e_{i}\mathrm{lg}e_{i}\\ e_{i}=a(i)/\sum_{i=1}^{N}a(i) \end{array}\right.$$ (17)

Where *i* = 1,2,⋯*N*, *N* is the number of signal sampling points; *E*_*e*_ is the envelope entropy; the envelope signal *E*_*e*_ after the Hilbert transform is *a*(*i*), and the normalized result is *e*_*i*_.

The BFO-PSO-VMD algorithm is implemented in the following steps:

1. The initialization parameters of the BFO-PSO algorithm set to the number of populations sizepop = 10, the number of convergence of populations Nc = 10, the number of swims Ns = 4, the number of replications Nre = 4, the number of dispersals Ned = 2, the probability of dispersal of populations Ped = 0.25 and the learning factor c1 = c2 = 1.4995.
2. Initialize the parameters $\left\{{\hat{u}}_{k}^{1}\right\}$、 $\left\{\omega_{k}^{1}\right\}$、 ${\hat{\lambda }}^{1}$、 *n* and the maximum number of iterations *m*_1_ in the VMD.
3. Bacterial positions are randomly initialized to generate a random vector of unit steps in any direction for each bacterium.
4. Convergence cycle operation. For each bacterium, the position is updated according to the flip, and if the position is better, it swum forward.
5. Update the local extremes of the individual bacteria and the global extremes of the bacterial swarm, and refer to the particle swarm algorithm formula provided in the literature [17] to update the direction of the return values of the bacteria.
6. Breeding operations. For a bacterial population that has undergone a chemotaxis cycle, each bacterium is ranked according to the cumulative and type of fitness, the less well adapted half of the bacteria are eliminated and the same population of bacteria is split from the better-adapted half, inheriting the position and characteristics of the parent bacterium.
7. Migration operations. After a cycle of propagation operations, a certain number of migrating bacteria is set, which is used to migrate the bacteria according to a certain migration probability, thus ensuring convergence of the algorithm and avoiding local extremes, to which the value of the combination [*k*, ɑ] is output.
8. Calculate ${\hat{u}}_{k}^{n+1}(\omega )$、 $\omega_{k}^{n+1}$、 ${\hat{\lambda }}^{n+1}(\omega )$ according to Eqs (8) to (10);
9. The convergence condition of Eq (11) is satisfied or *n*≥*m*_1_, the algorithm will stop iterating and output the value of the combination [*k*, ɑ]. The signal *f*(*t*) is decomposed into *k* modal components; otherwise *n* = *n*+1, return to step 8).

### R-LMS signal extraction

After VMD processing, the obtained IMFs contain mixed components and noise components. In order to better extract useful information from the mixed components, this paper proposes the R-LMS information extraction method. The cross-correlation coefficient R is introduced as a classification indicator. The IMFs generated by VMD are decomposed into mixed IMFs and noise IMFs. The noise IMFs are eliminated, while the mixed IMFs undergo secondary denoising using LMS, thereby maximizing the extraction of useful information.

The R value between the original signal and each IMF is calculated as a classification index. When the value of R is *R*≤0.2, it is considered to indicate a low correlation between the two variables and is defined as a noise IMF. The remaining IMFs are defined as mixed IMF. The formula for the cross-correlation coefficient R is as follows:

$$R\left(x(t),IMF_{k}\right)=\frac{Cov(x(t),IMF_{k})}{\sqrt{Var[x(t)]Var|IMF_{k}|}}$$ (18)

where *x*(*t*) is the original signal, *IMF*_*k*_ is the kth eigenmodal component of the VMD decomposition, *Cov*() is the covariance between *x*(*t*) and *IMF*_*k*_, and *Var*[] is the variance between *x*(*t*) and *IMF*_*k*_, *k* = 1,2⋯*n*.

After removing the noise components through pre-processing, the obtained hybrid IMFs are reconstructed. There are still quite a few noise components in the reconstructed signal, and considering the advantages of good convergence and high stability of the LMS algorithm [24], this paper performs secondary noise reduction through Least Mean Square Algorithm (LMS) to extract a more-effective components. The LMS is based on the most rapid descent method and adjusts the system weights along the negative gradient direction, replacing the mean squared error with the squared error. Its iterative formula is as follows.

$$y(n)=W^{T}(n)X(n)$$ (19)

e(n)=d(n)−y(n) (20)

W(n+1)=W(n)+2μe(n)X(n) (21)

where: *X*(*n*) is the input mixed signal, *y*(*n*) is the output signal, *e*(*n*) is the error signal, *d*(*n*) is the desired signal, *μ* is the iteration step factor, and for the algorithm to be convergent, the step factor *μ* is taken to satisfy.


$$0\leq \mu \leq 1/\lambda_{\max}$$ (22)

*λ*_max_ denotes the maximum eigenvalue of the autocorrelation matrix of *X*(*n*).

## Improved VMD-BPNN detection algorithm

**Fig 2:**
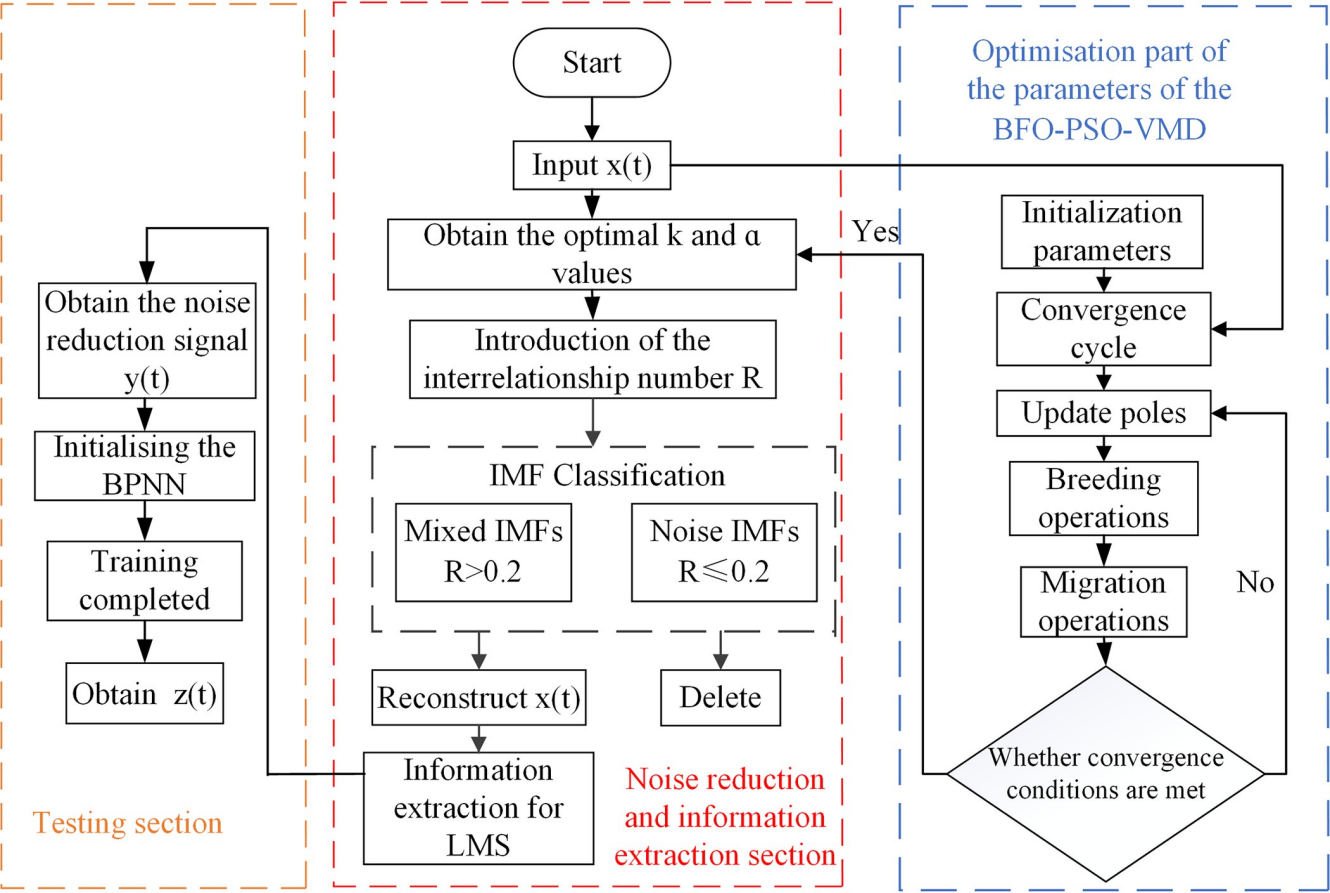
Flow chart of the algorithm structure of this paper.

The signal-to-noise ratio is greatly improved by the BFO-PSO-VMD-R-LMS pre-processing of the residual current signal. Combining the processed signal with the BPNN to form the BFO-PSO-VMD-LMS- BPNN detection algorithm can further improve the detection accuracy of the algorithm. The flow chart is shown in Fig 2.

The process is as follows.

1. Decomposition of the original signal into K IMFs components by means of an optimization section.
2. Calculation of the interrelationship number R between the individual IMF components and the original signal, and classification of the IMF according to the value of R.
3. The noisy IMFs are rounded off and the mixed IMFs are reconstructed to obtain the signal *x*(*t*) for information extraction and the pre-processed signal *y*(*t*).
4. Initialization of the BPNN followed by detection of the pre-processed signal, and then training of the BPNN using the reconstructed signal to obtain a neural network satisfying the detection accuracy.
5. The trained BPNN processes the reconstructed signal to obtain the final detection signal *z*(*t*). Based on the detected signal, the prediction of the residual current can be achieved.

The BFO-PSO-VMD-R-LMS-BPNN detection pseudocodes are shown in Table 1.

**Table 1:** BFO-PSO-VMD-R-LMS-BPNN detection pseudocodes.

BFO-PSO-VMD-R-LMS-BPNN detection		
**Begin**		
**Input:** Nc = 10, Nre = 4, Ped = 0.25 c1 = c2 = 1.4995. **Output:** A collection of signal decomposition modes u The spectral range of the model u_hat	**Input: [***k*, ɑ**], [**u, u_hat**],** filter order L = 20, convergence factor Mu = 0.05. Input signal *X*_*n*_, Desired signal *d*_*n*_, Output signal *y*_*n*_	
**1**: **function: [**u, u_hat] = VMD (Fv, ɑ, k,)	**9.function $X_{n}\leftarrow R(u,IMF_{k})=Cov(u,IMF_{k})/\sqrt{Var[u]Var|IMF_{k}|}$**	
**2**. **Initial population** for i = 1: sizepop	**10.end for output** *X*_*n*_	
**3**. **Calculate the fitness value** $J(i,j,k,l)\leftarrow J(i,j,k,l)+J_{cc}[\theta^{i}(j,k,l)+P(j,k,l)]$	**11.function LMS** (*X*_*n*_, *d*_*n*_,L, Mu) $W(n+1)=W(n)+2\mu e(n)X_{n}$	
**4**. **Iterative optimization** For a = 1: Ned; For b = 1: Nre; For c = 1: Nc;	**12. if** 0≤*μ*≤1/*λ*_max_	
$\theta^{i}(j+1,k,l)\leftarrow X_{i}^{k}+[\omega V_{i}^{k}+c_{1}r_{1}(P_{i}^{k}-x_{i}^{k})+c_{2}r_{2}(P_{g}^{k}-X_{i}^{k})]\varphi (j)$	**13.end for Output (*y***_***n***_ **)**	
**5.** if $\sum_{k+1}^{k}{\left\|{\hat{u}}_{k}^{n+1}-{\hat{u}}_{k}^{n}\right\|}_{2}^{2}/{\left\|{\hat{u}}_{k}^{n}\right\|}_{2}^{2}<\varepsilon_{1}$	**14. Input:** the learning rate = 0.01; Training times = 1000 **function BPNN (*y***_***n***_**)**	
**6. end for**	**15.** $\alpha_{h}\leftarrow f_{1}(y_{n})$ $z_{n}\leftarrow f_{2}(\alpha_{h})$	
**7. Output [***k*, ɑ**], [**u, u_hat**]**	**16. end for Output(*z***_***n***_ **)**	
**8. end function BFO-PSO-VMD**	**17.END**	

### Analysis of human simulation experiments

**Fig 3:**
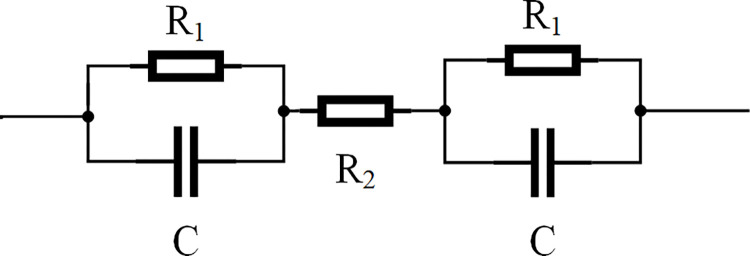
Freiberg plus human equivalent circuit model.

To verify the effectiveness of BFO-PSO-VMD-LMS-BPNN in this paper, the human equivalent circuit model of H. Freiberger was firstly constructed in MATLAB/Simulink platform for human electrocution simulation experiments [25,26], and the equivalent circuit diagram is shown in Fig 3.

According to the experimental results of H.Freiberger, the skin impedance R_1_ is 2000 Ω at a single-phase AC voltage of 220V, the skin capacitance C is 2×10^−8^ F, and the internal body impedance R_2_ is 500 Ω. Therefore, a single-phase alternating current of 50 Hz and a voltage of 220 V is applied to the human equivalent circuit, starting at 0.05s and ending at 0.16s. Forty-five sets of residual current simulated signals from the human body in the event of an electrical shock fault were obtained, with each set of data intercepting 8 cycles of the signal waveform respectively, for a total of 800 sampling points. To better simulate the condition of human electrocution, Add 10dB, 5dB, and 0dB of Gaussian white noise to the simulated signal, 15 sets of each. The experimental parameters were set as shown in Table 2.

**Table 2:** Experimental setup parameters.

Parameters	Set values	Parameters	Set values
the number of populations	10	the learning factor	1.4995
convergence of populations	10	iterations number	80
the number of swims	4	count of independent runs	10
the number of replications	4	the learning rate	0.01
the number of dispersals	2	Training times	1000
the probability of dispersal	0.25	Environmental noise	0dB,5dB,10dB

### Simulation signal detection

**Fig 4:**
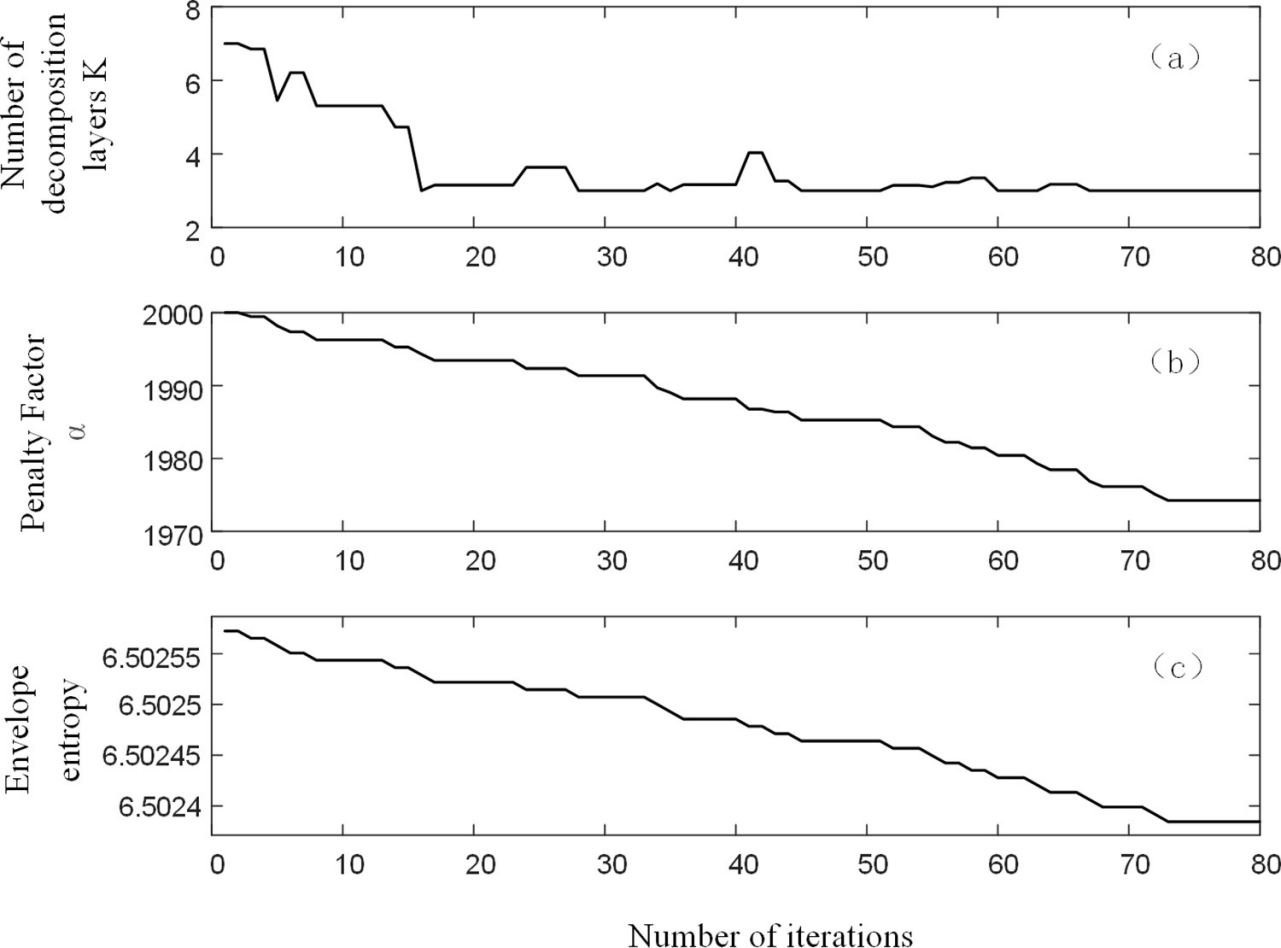
Iterative plots of k, ɑ, envelope entropy.

**Fig 5:**
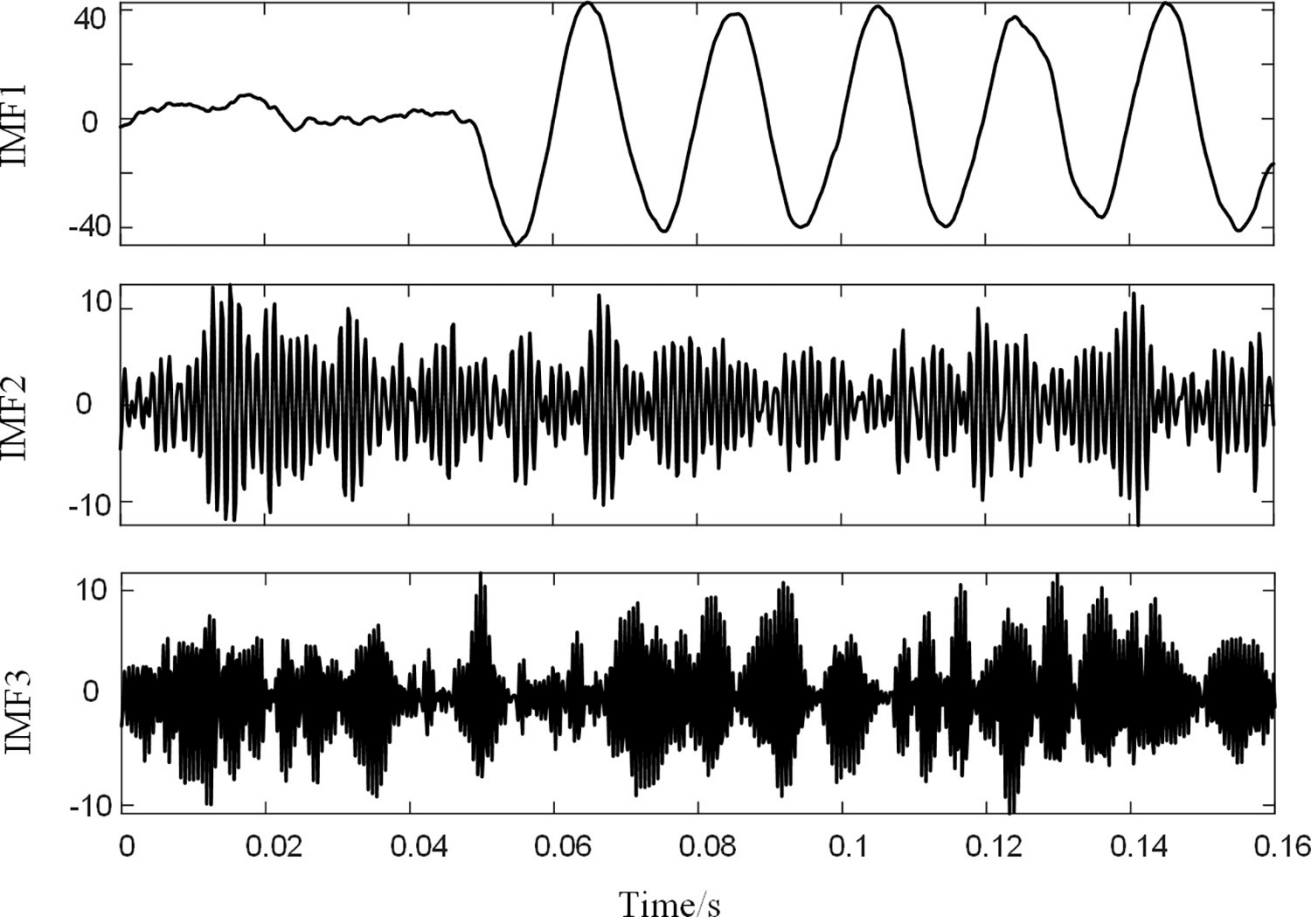
IMFs component.

Taking a certain set of residual current analogue signals with a signal-to-noise ratio of 10 dB as an example, BFO-PSO-VMD was used to denoise the analogue signals, with each set of signals run independently for 10 times and a maximum number of 80 iterations. The optimal combination of parameters [*k*, ɑ] for this group of signals was obtained. Fig 4 shows the iterative search process for the group. Analysis of Fig 4(C) shows that the group evolved to a local minimum entropy value of 6.5023 by the 72nd generation. the corresponding optimal combination of parameters is [3,1974], as shown in Fig 4(A) and 4(B). After decomposing this set of residual current signals using the optimal combination of parameters, the results are shown in Fig 5.

**Fig 6:**
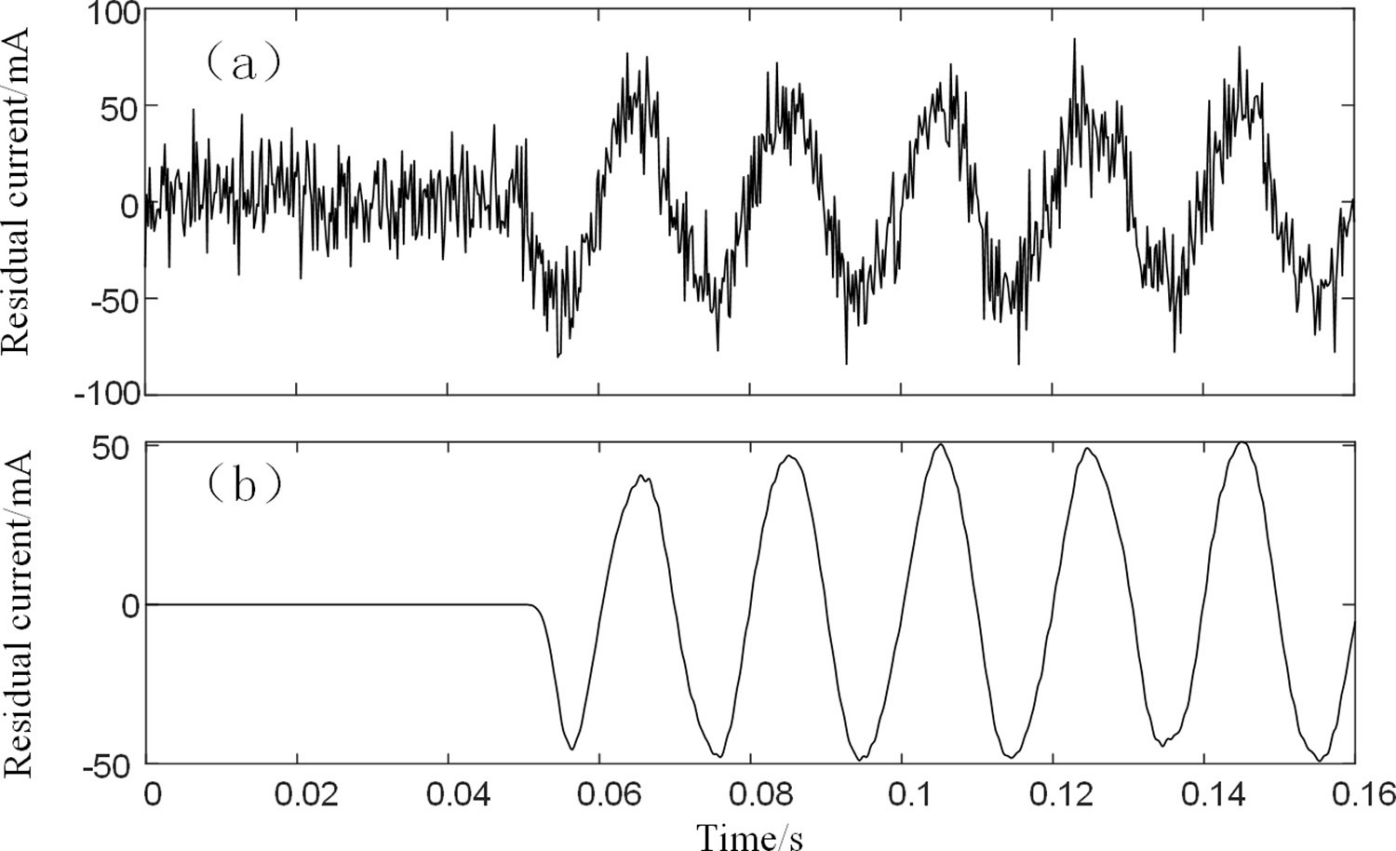
(a) Simulated signal (b) Reconstructed signal.

After calculating the interrelationship number R, the interrelationship indices of the three IMFs with the simulated signals are 0.8855, 0.2592, and 0.0081, respectively. According to the rules for the selection of noisy IMFs and mixed IMFs, IMF3 is the noisy IMF, and the remaining IMFs components are reconstructed and the reconstructed signal is filtered using LMS. Where the order of the LMS filter is 3, the initial value of the filter is set to 0 and the initial error is 0.01. The signal after LMS processing is shown in Fig 6.

As seen in Fig 6, the BFO-PSO-VMD-R-LMS algorithm can reconstruct the signal well when the signal-to-noise ratio is 10dB. The correlation coefficient between the reconstructed signal and the original signal is calculated to be 0.937, which meets the criteria for continued residual current detection.

### BPNN Detection

**Fig 7:**
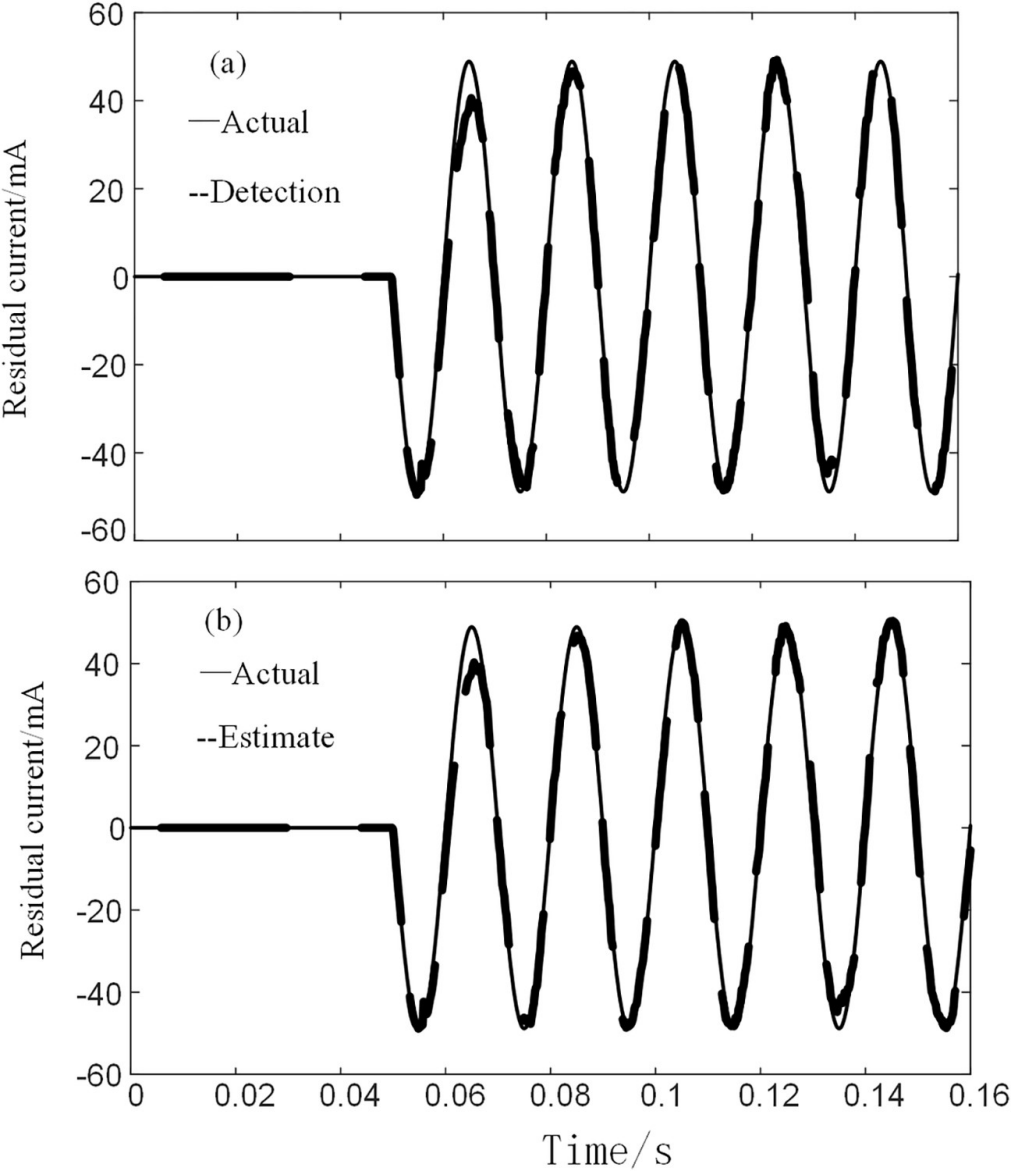
BPNN detection and prediction. (a) Actual and detected values (b) Actual and predicted values.

The residual current simulated signal processed by the BFO-PSO-VMD-R-LMS algorithm was well recovered, so the reconstructed signal was fed into the BPNN of the training number for detection and the signal was predicted by the data of the first three cycles. The residual current detection and prediction waveforms of the simulated signal are shown in Fig 7.

As can be seen from Fig 7, the actual value and the detected value curve basically coincide with the signal detected by the BPNN. The interrelationship between the actual and detected values is calculated to be 0.9567; while the interrelationship between the actual and predicted values is 0.8939, which can satisfy the detection accuracy of the residual current device.

### Comparison of different algorithms

When the signal-to-noise ratio is 10dB, 5dB, and 0dB, in order to illustrate the superiority of BFO-PSO-VMD-LMS- BPNN, a comparison is made with VMD-LSTM and N-LMS in terms of operation time and root mean square error signal-to-noise as well as signal-to-noise ratio as follows.

$$SNR=10\log_{10}\frac{{\left\|S_{1}\right\|}^{2}}{{\left\|S_{2}-S_{1}\right\|}^{2}}$$ (23)

where S_1_ is the original pure signal and S_2_ is the processed noise reduction signal after noise addition.

$$RMSE=\sqrt{\frac{1}{m}\sum_{i=1}^{m}{\left(y_{i}-{\hat{y}}_{i}\right)}^{2}}$$ (24)

where m is the number of samples, *y*_*i*_ is the signal before noise reduction, and the signal after noise reduction.

$$R^{2}=1-\frac{\sum {\left(Y_{a}-Y_{p}\right)}^{2}}{\sum {\left(Y_{a}-Y_{m}\right)}^{2}}$$ (25)

where *Y*_*a*_ is the sum sample value, *Y*_*p*_ is the predicted value and *Y*_*m*_ is the sample mean.

The specific data is shown in Table 3.

**Table 3:** Comparison of the detection results.

Environmental noise	Indicators	VMD-LSTM	N-LMS	BFO-PSO-R-LMS-BPNN
10dB	*t/s*	1.8213	1.7961	**1.7255**
	SNR	12.5826	13.1522	**15.1161**
	RMSE	0.2828	0.2646	**0.2449**
	R^2^	0.9101	0.9759	**0.9847**
5dB	*t/s*	1.6876	1.6356	**1.6182**
	SNR	11.1692	12.8435	**14.2296**
	RMSE	0.2583	0.2645	**0.2508**
	R^2^	0.9631	0.9745	**0.9807**
0dB	*t/s*	1.7825	1.7156	**1.6831**
	SNR	7.1792	6.4365	**8.5811**
	RMSE	0.2583	0.2721	**0.2573**
	R^2^	0.9106	0.9050	**0.9384**

Analysis of Table 3 shows that the algorithm BFO-PSO-VMD-LMS-BPNN outperforms the other two algorithms in terms of speed, RMSE, SNR, and R^2^ for the same noise. The speed of the operation and the root mean square error were improved, but the difference was not significant. However, in terms of SNR ratio, they improved over the remaining two algorithms by 20.1%, 11.9%; 27.4%, 10.8%; 33.32%, and 19.52% respectively. The change of R^2^ is more obvious at 0dB and is 3.05% and 3.69% higher than the rest of the algorithms respectively, which shows that the noise reduction effect of this paper is better and the detection accuracy is higher.

## Analysis of actual measurement data

**Fig 8:**
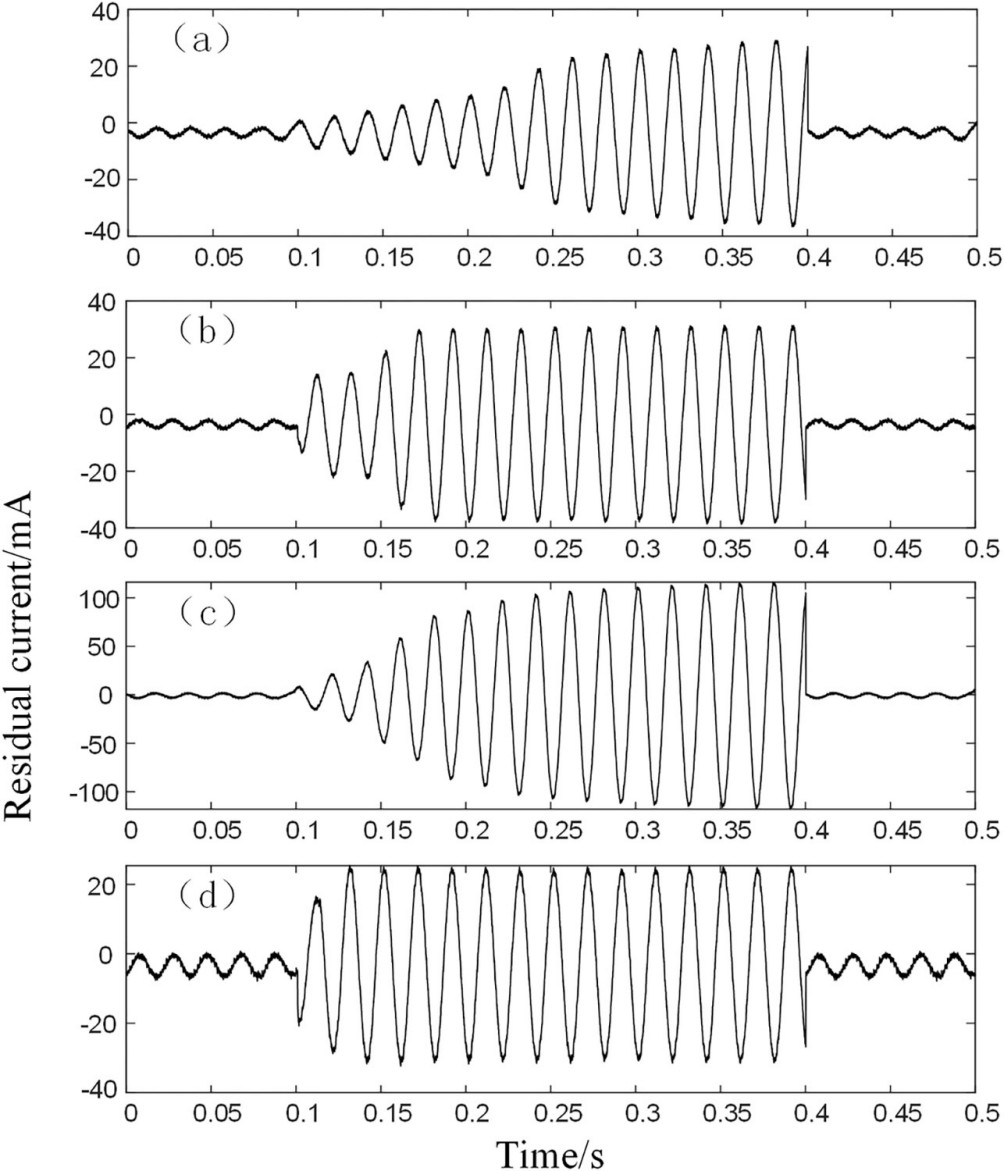
Residual current real measurements for different types. (a) Grass (b) Poplar (c) Wetland (d) Concrete.

Residual current signals are collected in a variety of situations using a laboratory-built residual current detection platform. The experimental platform is equipped with a voltage of 220V and a frequency of 50Hz AC. The experimental data is sampled for 0.5s at a time and is set to fail at 0.1s and disconnect at 0.4s. The oscilloscope has a sampling frequency of 10 kHz and acquires 15 cycles of valid data. Residual current signals were collected for a total of four fault types: poplar, grass, concrete, and wet ground. In the different types Under different types, 20 sets of experimental data were acquired by Tektronix MDO3024 mixed domain oscilloscope in high-resolution sampling mode respectively. The residual current waveforms for the different fault types are shown in Fig 8.

### Measured data noise reduction

**Fig 9:**
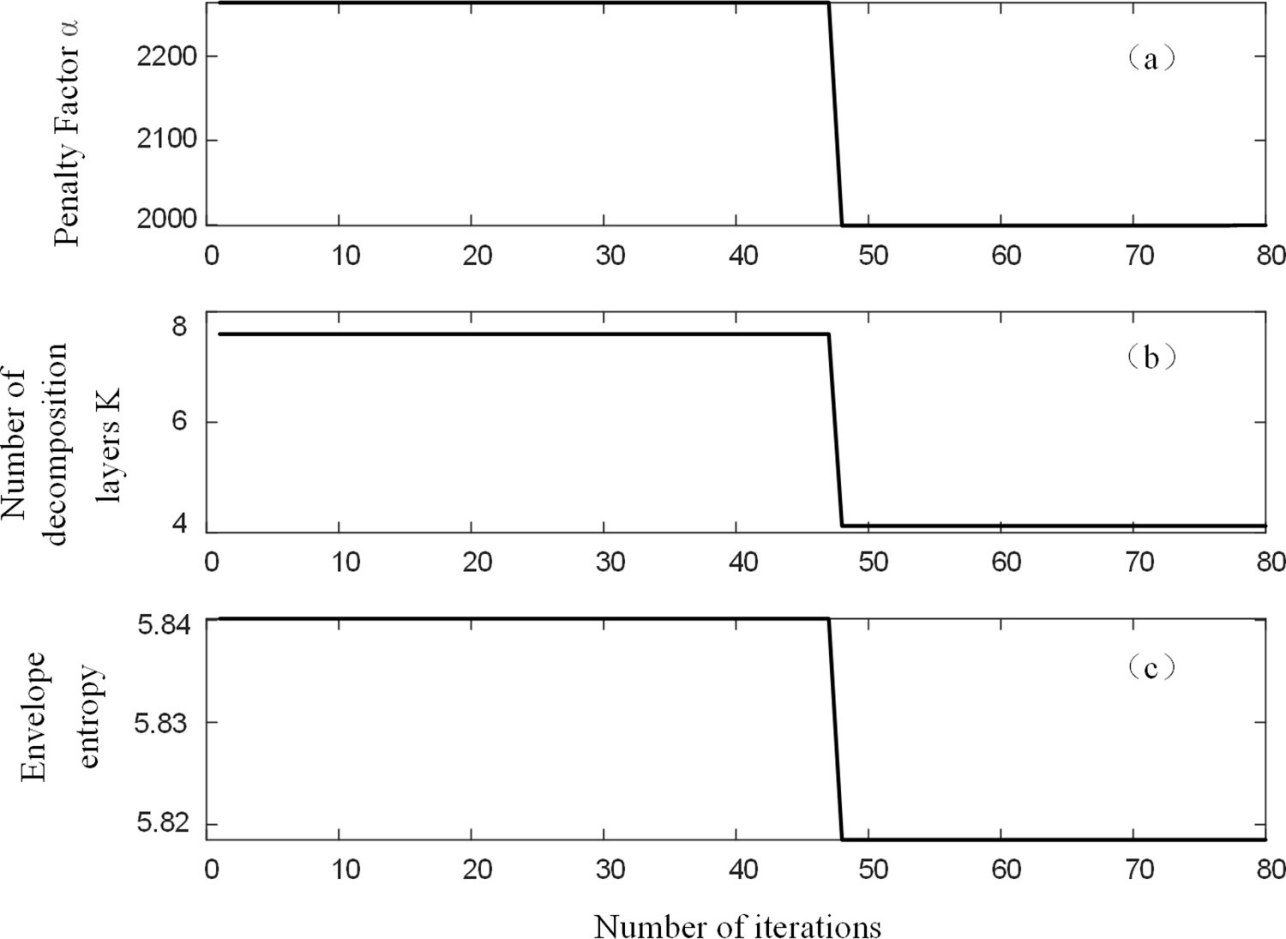
Iterative plots of k, ɑ, envelope entropy.

As an example, the residual current signal of a line single-phase earth in a wetland situation is simulated by adding Gaussian white noise with a signal-to-noise ratio of 10 dB, as the oscilloscope comes with a filtering function in high-resolution mode. The VMD optimized parameters are shown in Fig 9.

**Fig 10:**
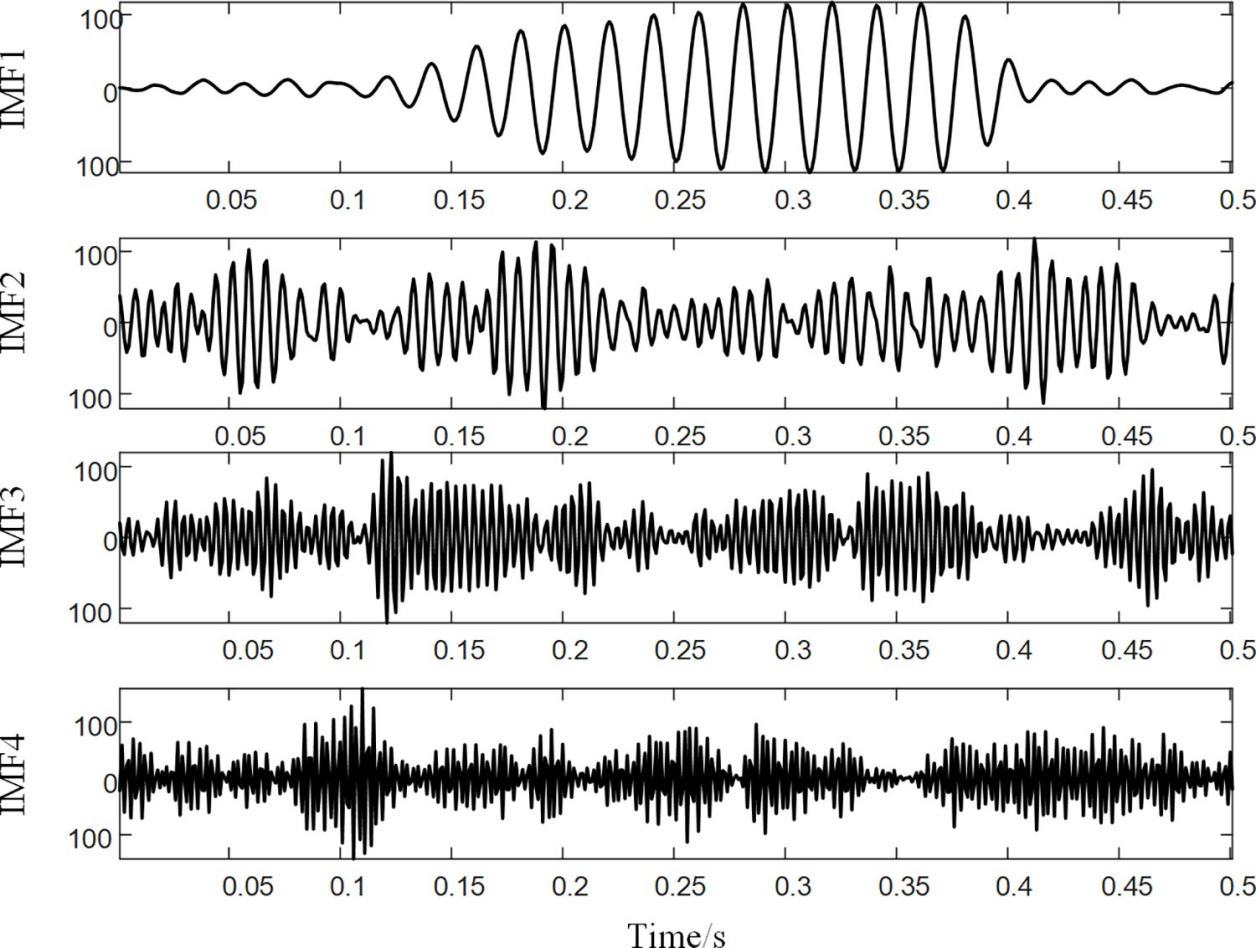
IMFs component.

Analysis of Fig 9(C) shows that the population evolved to the 48th generation to obtain a local minimum entropy value of 5.8185. The corresponding optimal combination of parameters is [4,2000], as shown in Fig 9(A) and 9(B). After VMD decomposition of the residual current signal with the addition of a signal-to-noise ratio of 10 dB, the results are shown in Fig 10.

**Fig 11:**
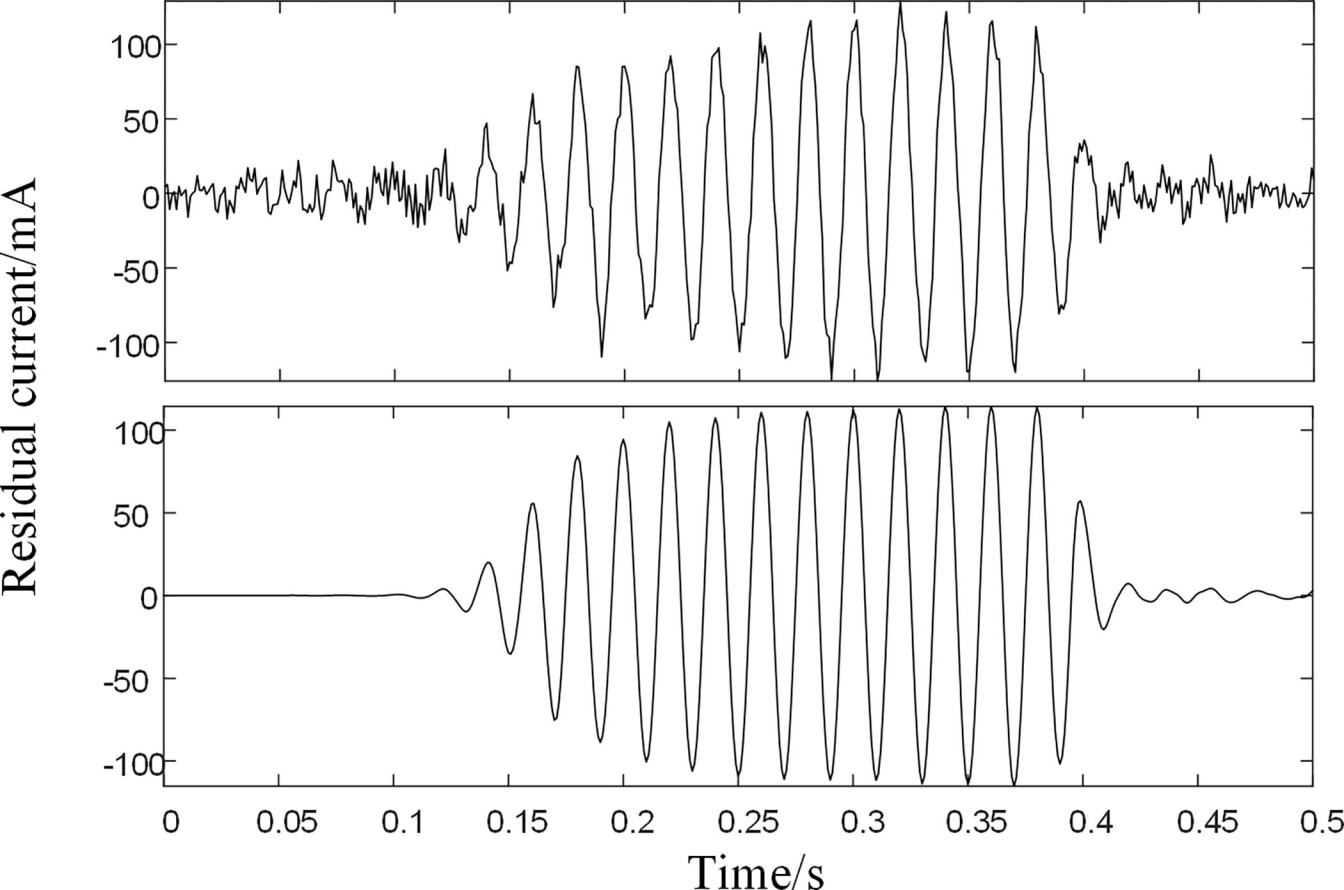
Comparison of the original simulated signal and the noise reduction signal. (a) Original signal (b) Noise reduction signal.

After calculating the mutual correlation number R, the mutual correlation indices of the four IMFs and the simulated signal are 0.9593, 0.2289, 0.08921, and 0.03981, respectively. According to the filtering rule, IMF1 and IMF2 are defined as mixed components and IMF3 and IMF4 are defined as noise components, and IMF1 and IMF2 are reconstructed and subjected to secondary noise reduction by the LMS algorithm. The noise reduction results are compared with the simulated signal as shown in Fig 11.

**Fig 12:**
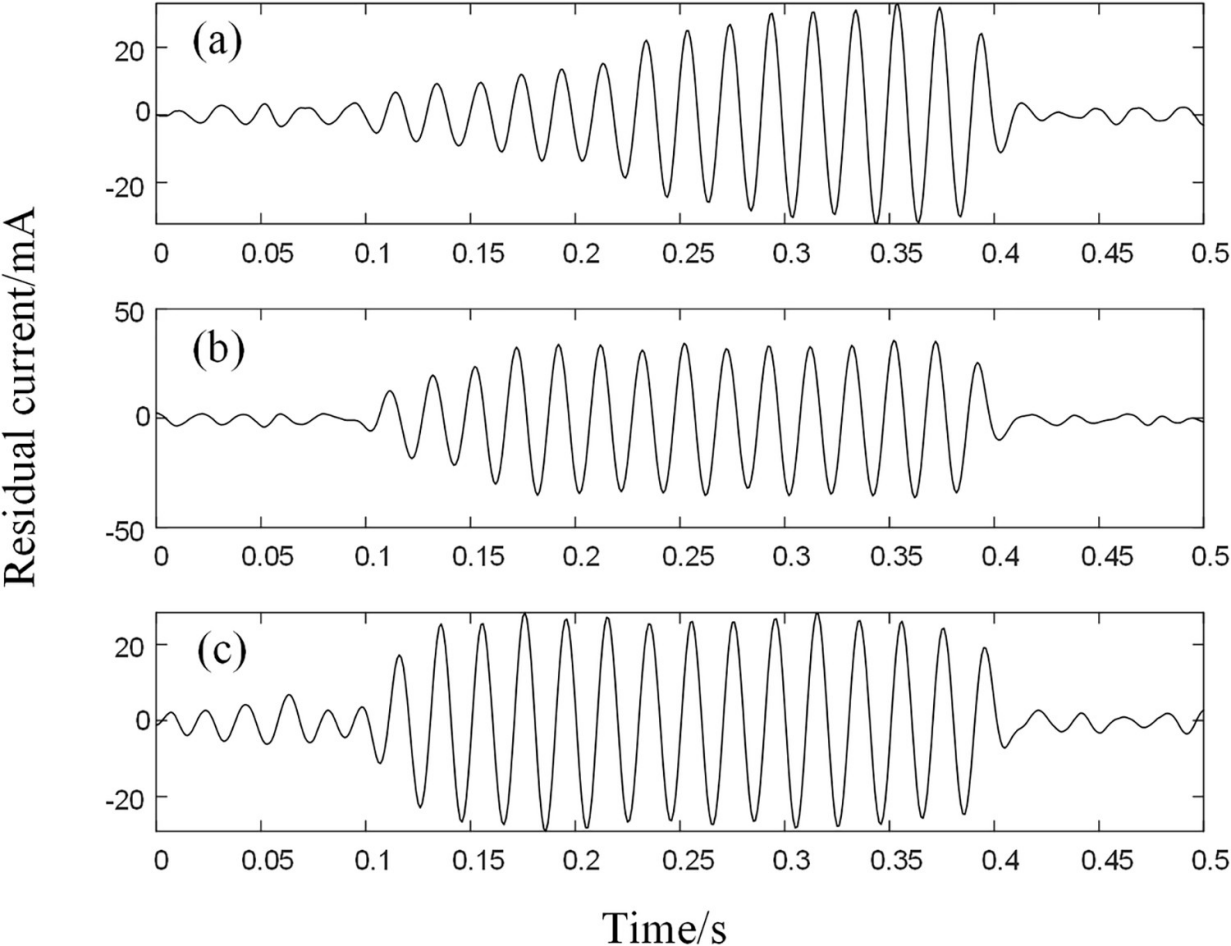
LMS noise reduction signal (a) Poplar tree (b) Grass (c) Concrete ground.

Additional noise reduction plots processed by the algorithm are shown below. As can be seen from Figs 11 and 12, the four fault signals are well recovered after BFO-PSO-VMD-R-LMS processing.

### BPNN detection

As an example, two types of faults occur in the case of single-phase direct grounding of lines in the case of poplar trees represented by plant electrocution and in the case of wetland, the experimentally collected residual current signal has 15 valid cycles with a total of 3,000 sampling points. The algorithm of this paper is used to detect the fault signal and to make a prediction of the corresponding signal by the first 10 valid cycles.

**Fig 13:**
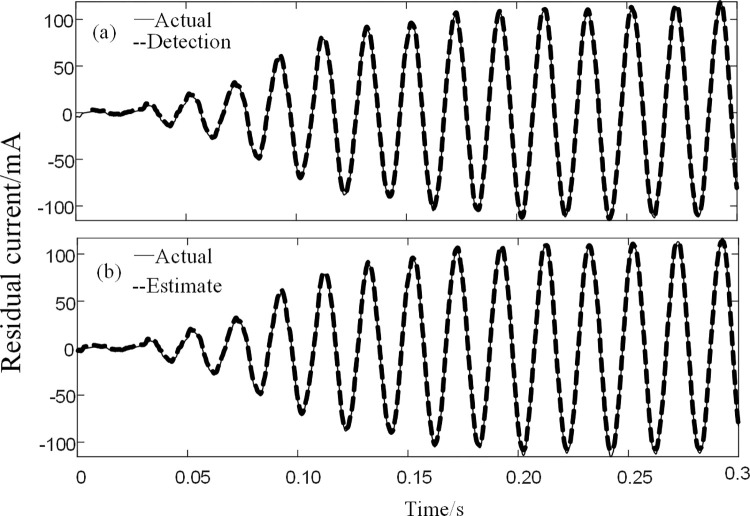
BPNN plant electrocution current detection. (a) Actual and detected values (b) Actual and predicted values.

**Fig 14:**
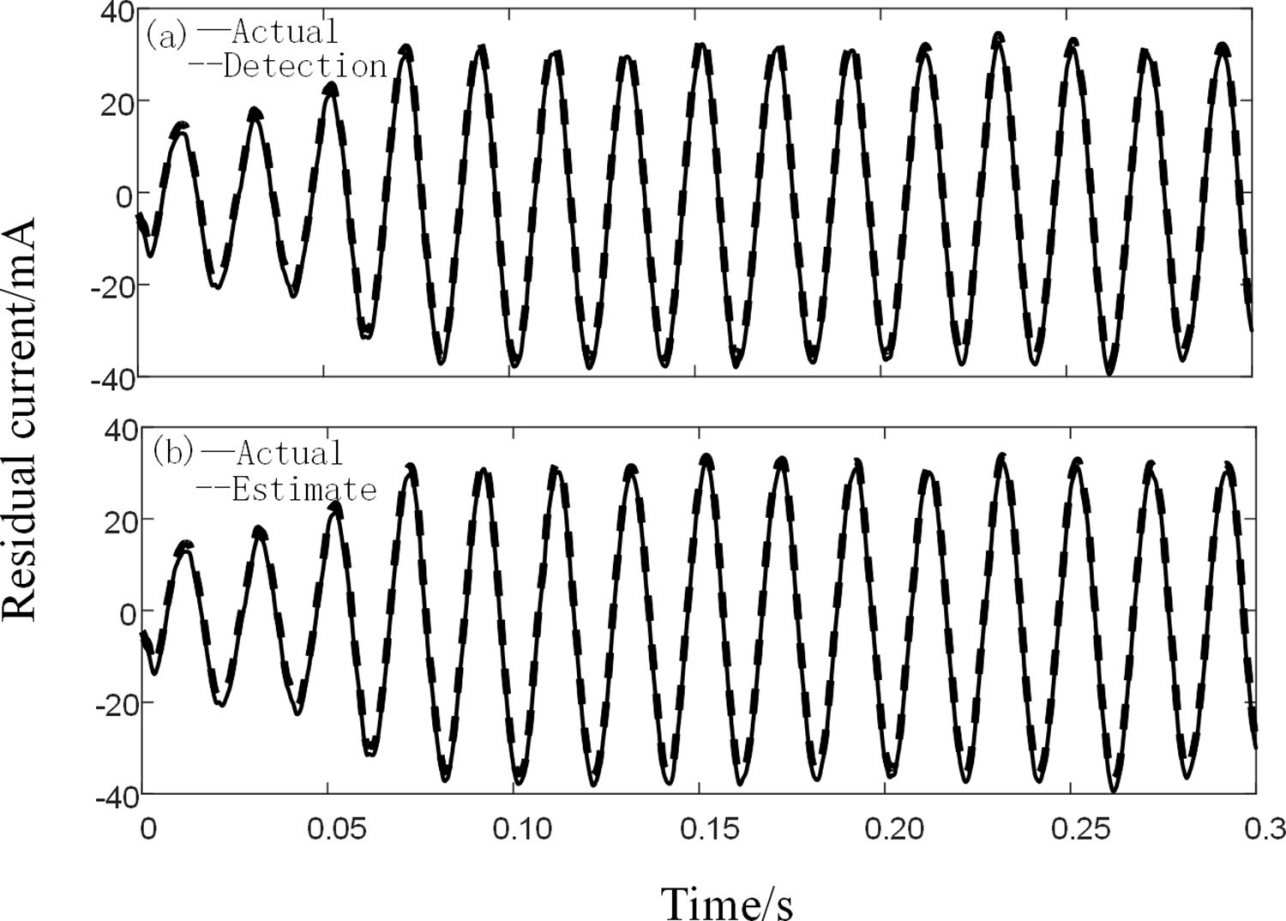
Single-phase direct grounding of BPNN line. (a) Actual and detected values (b) Actual and predicted values.

The detected and predicted waveforms for the two different types of residual current are shown in Figs 13 and 14 respectively.

From Figs 13 and 14, it can be concluded that: the error of BFO-PSO-VMD -R-LMS-BPNN for the detection of two fault residual current signals is small, the detection error is 4.96% and 3.35% respectively, while the prediction accuracy of the two sets of data by BPNN for the fault residual current signal is 91.37% and 92.56% respectively, which can meet the residual The accuracy of the residual circuit detection device can be satisfied.

### Comparison of different algorithms

To further demonstrate the advantages of the BFO-PSO-VMD-R-LMS-BPNN processing of measured residual current signals, it was compared with both VMD-LSTM and N-LMS algorithms and the results are shown in Table 4. The results were statistically analyzed using the Kolmogorov-Smirnov test (K-S test) as shown in Table 5.

**Table 4:** Comparison of the detection results.

Environmental noise	Indicators	VMD-LSTM	N-LMS	BFO-PSO-R-LMS-BPNN
10dB	t/s	2.1568	1.9836	**1.9118**
	SNR	14.0221	14.3728	**16.3018**
	RMSE	0.5606	0.4583	**0.4359**
	R^2^	0.9256	0.9312	**0.9627**
5dB	t/s	2.2356	2.1968	**2.0867**
	SNR	10.4925	11.1261	**13.6791**
	RMSE	0.4561	0.4796	**0.4494**
	R^2^	0.9064	0.9109	**0.9373**
0dB	t/s	2.2689	2.1648	**2.1559**
	SNR	6.1809	6.7623	**7.7300**
	RMSE	0.4587	0.4853	**0.4567**
	R^2^	0.8703	0.8926	**0.9166**

**Table 5:** Kolmogorov-Smirnov test data analysis table.

Algorithm name	Average value	Standard deviation	skewness	kurtosis	p-value
LSTM	3.461	4.463	1.731	2.046	-----
BPNN	4.002	5.53	1.662	1.468	-----
N-LMS	3.564	4.674	1.682	1.732	-----
LSTM pairs BPNN	-0.541	1.142	-1.662	1.567	0.021**
N-LMS pair BPNN	-0.438	0.899	-1.804	2.101	0.018**

Note

***、** arepresents a significance level of 1% and 5%, respectively.

As shown in Tables 4 and 5, the algorithm proposed in this paper outperforms the other two algorithms in terms of speed, SNR, R^2^, and RMSE for the same level of noise. The signal-to-noise ratio (SNR) is an important indicator of signal purity, and a higher SNR indicates better noise reduction. Therefore, the higher the SNR, the better the noise reduction. The goodness-of-fit represents the degree of fit of the regression line to the observed values, and a higher signal purity leads to higher detection accuracy of the BP neural network. By combining the K-S test to analyze the results, it was found that the significance p-values were 0.021 and 0.018 when this algorithm was compared with VMD-LSTM and N-LMS, respectively, indicating statistical significance at the level of experimental data. These results demonstrate that the proposed algorithm has higher detection accuracy and better performance.

## Conclusion

This paper proposes the BFO-PSO-VMD-R-LMS-BPNN detection method for detecting fault residual currents in low-voltage networks. The optimal combination of parameters is obtained by BFO-PSO for the VMD parameters [*k*, ɑ] seeking and combined with the interrelationship number R and LMS for classification and reconstruction, which avoids noise interference in the detection using the original signal and ensures the recognition accuracy of the residual current signal while combining with BPNN for the detection and prediction of the pre-processed signal, which has higher accuracy and robustness.

Compared with the N-LMS and VMD-LSTM methods, the proposed algorithm achieves better signal processing in a shorter time while ensuring the accuracy of the residual current signal. Simulation and experimental data demonstrate that the proposed algorithm outperforms the other two algorithms in terms of R^2^, SNR, and RMSE. In actual measurements, the SNR is improved by an average of 23.87% and 16.89% compared to the VMD-LSTM and N-LMS algorithms, respectively, and R^2^ is improved by an average of 4.24% and 2.99%, with little difference in operation speed and RMSE. The proposed algorithm exhibits high accuracy and can be used in adaptive RCD to achieve instantaneous response to electric leakage or shock accidents. However, the algorithm in this paper does not differ significantly from the remaining two algorithms in terms of speed, and there are possibilities for improvement, for example by adjusting structural parameters and exploring other methods to further improve the details of the model. Finally it is recommended that future research can incorporate prediction data, extract eigenvalues from residual current signals and systematically classify residual currents to better identify fault types and develop appropriate measures to ensure personal safety.

## Supporting information

S1 Data (MAT)

S2 Data (MAT)

S3 Data (MAT)

S4 Data (MAT)

S5 Data (MAT)
